# Influence of post hatch dietary supplementation of fat on performance, carcass cuts and biochemical profile in Ven Cobb broiler

**DOI:** 10.14202/vetworld.2015.187-191

**Published:** 2015-02-16

**Authors:** Komal Prasad Rai, M. K. Gendley, S. P. Tiwari, Tarini Sahu, Surendra Kumar Naik

**Affiliations:** Department of Animal Nutrition, College of Veterinary Science and Animal Husbandry, Anjora, Chhattisgarh Kamdhenu Vishwavidyalaya, Durg, Chhattisgarh, India

**Keywords:** biochemical profile, broiler, carcass cuts, fat, performance, post hatch

## Abstract

**Aim:**

The present experiment was conducted to study the effect of post hatch dietary fat supplementation on performance of broiler chicken.

**Materials and Methods:**

A total of 120 day-old Ven Cobb broiler chicks were randomly assigned to 4 treatment groups of 30 chicks in each (three replicates of 10 birds/treatment). The trial lasted for 35 days. The experimental design was a completely randomized design. Four types of diet were formulated for 1^st^ week: T_1_, T_2_, T_3_ and T_4_ contained control diet with no added fat, 2.5, 5 and 7.5% fat, respectively. After 1^st^ week post-hatch period chicks were fed *ad libitum* with the normal basal diet as per Bureau of Indian Standard recommendations till completion of the experiment (8-35 days).

**Results:**

Significantly higher (p<0.05) body weight and improved feed conversion ratio (FCR) was recorded in birds fed 5% dietary fat at the end of the experiment whereas, feed intake was not significantly affected. Significantly (p<0.05) higher dressed weight was observed due to 5% fat supplementation than other groups whereas, it was not significant for other carcass cuts.

No significant differences were observed in moisture, protein and lipid content of breast and thigh muscle of broiler due to supplemented fat whereas, 2.5% dietary fat significantly (p<0.05) increase the serum HI titer on day 28^th^.

In biochemical profile, higher serum albumin (g/dl) was recorded due to 5% fat supplementation whereas other biochemical components did not show any significance difference among treatments.

**Conclusion:**

It may be concluded that supplementation of fat in broilers diet improves the overall FCR, dressing percentage and gain more body weight.

## Introduction

In the developing poultry embryo, the sole energy source is yolk. Yolk is utilized in newly hatched chick either by direct transfer to circulation or by transport through the yolk stalk into the small intestine. Anti-peristaltic movements transfer the yolk to the proximal part of the small intestine whereas, pancreatic lipase digest the acyl lipids. Thus, oleic acid is highly absorbed close to hatch. In contrast, hydrophilic compounds such as glucose and amino acids are not well absorbed in yolk rich medium. Absorption of glucose and amino acids increases with age and with the development of hydrophilic conditions in the intestinal lumen [1]. During 1^st^ week post hatch nutrient utilization is gradually increased. Therefore this stage of development is very crucial for modern broiler industry. The objective, of feeding the 1^st^ week post-hatch diet, is to provide the nutrient dense ration to the birds [2]. Researchers have shown that final growth performance of chicken had the linear correlation with their 1^st^ week of rearing [3]. Delayed access to feed adversely affects chicks’ performance [4].

Therefore, the present investigation was carried out to determine the influence of post hatch dietary fat on performance, carcass yield and biochemical profile of broiler chicken.

## Materials and Methods

The experiment was conducted in the Department of Animal Nutrition, College of Veterinary Science and Animal Husbandry, Anjora, Durg (C.G.).

### Ethical approval

The present study was approved by Institutional Animal Ethics Committee.

### Experimental design

A total of 120 day-old Ven Cobb broiler chicks were randomly assigned to 4 treatment groups of 30 chicks in each (three replicates of 10 birds/treatment) following completely randomized design. The trial lasted for 35 days. Chicks were reared in deep litter system under standard housing and management condition. Birds were vaccinated against Ranikhet disease (F strain) and Infectious Bursal disease on day 7 and day 14, respectively. A booster dose for Ranikhet disease was given on day 28. Broiler starter and finisher rations were offered to the birds in mash form. Fresh and clean drinking water was always made available to the birds during the study. Artificial light was provided during night hours in order to extend 24 h photoperiod.

### Treatments

Four types of diet were formulated for 1^st^ week: T_1_, T_2_, T_3_ and T_4_ contained control diet with no added fat, 2.5, 5 and 7.5% fat, respectively. Ingredient and nutrients composition of experimental starter diets for chicks at 0-7 days old were presented in Tables-1 and -2 respectively. After 1^st^ week post-hatch period chicks were fed *ad libitum* with the normal basal diet as per Bureau of Indian Standard [5] recommendations till completion of the experiment (8-35 days).

**Table-1:** Ingredient composition of experimental broiler starter (0-7 d) diet (on % DM basis).

Feed ingredients	Control	2.5% fat	5% fat	7.5% fat
Yellow maize	50.5	48.5	38	28.5
Soya DOC	41	31.5	33.5	34.5
Maize gluten	-	9	6.75	5.5
Deoiled rice bran	-	0	8.25	15.5
Soyabean oil	-	2.5	5	7.5
Dicalcium phosphate	3	3	3	3
Limestone powder	1.2	1.2	1.2	1.2
DL-Methionine	0.7	0.7	0.7	0.7
Lysine	1.4	1.4	1.4	1.4
Soda-bi-carb	0.3	0.3	0.3	0.3
Choline chloride	0.14	0.14	0.14	0.14
Salt	0.8	0.8	0.8	0.8
Premix*	0.96	0.96	0.96	0.96

* Trace mineral premix mg/kg diet: Mg 300, Mn 55, Fe 56, Zn 30, Cu 4, vitamin premix per kg diet: Vitamin A 8250IU, vitamin K 1 mg, vitamin E 26.84 mg, vitamin B_1_ 2 mg, vitamin B_2_ 4 mg, vitamin B_12_ 100 mg, Niacin 60 mg, pantothenic acid 10 mg; choline 500 mg and 30 ppm salinomycin (Coxistac 12%), 55 ppm bacitracin methylene di salicyclate (BMD110), DM=Dry matter

**Table-2:** Chemical composition of experimental broiler starter (0-7 d) diet (on % DM basis).

Diet (%)	Moisture (%)	CP (%)	CF (%)	EE (%)	ME (kcal/kg)	Total ash (%)	AIA (%)	NFE (%)	Ca (%)	p (%)
Control	9.78	23.01	3.99	2.67	2803.00	10.30	1.35	60.03	1.14	0.57
2.5 fat	10.74	23.03	2.57	6.44	2799.00	10.25	1.51	57.71	1.27	0.54
5 fat	10.41	23.02	3.44	8.14	2803.00	10.93	1.53	54.47	1.26	0.55
7.5 fat	9.90	23.01	4.31	10.22	2807.00	11.10	1.52	51.36	1.23	0.59

EE=Ether extract, CF=Crude fiber, CP=Crude protein, NFE=Nitrogen free extract, DM=Dry matter

### Measurements

Feed intake and body weight were measured and then feed conversion ratio (FCR) was calculated weekly. At 35 day of age, 3 birds of identical body weight from each replicate were sacrificed. The birds were fasted overnight, bled, defeathered and eviscerated with sufficient care. Different carcass parameters such as defeathered weight, eviscerated weight, and weight of liver, heart and gizzard were recorded. Each of carcass components such as breast, legs, wings and back with neck was recorded and presented as a percentage of live body weight.

### Protein and lipid analysis

On day 35, total lipid in breast and thigh ­muscles was determined as per the procedure of Bligh and Dyer [6] with minor modification. Moisture and protein were subjected to analysis as per AOAC [7].

### Humoral immunity

Humoral immune response was assessed by hemagglutination inhibition (HI) test. The blood was collected from 3 birds of each replicate in non-heparinized clean test tube on 28 and 35 day of the experiment. The serum was collected as per standard procedure and mean serum HI titer were evaluated against Newcastle disease virus according to the method described by Allan and Gough [8].

### Serum biochemical profile

Blood was collected from the jugular vein in non-heparinized, and clean test tubes from 3 birds each replicate on 35 day of the experiment. The serum was separated as per the standard procedure and stored under deep freezing temperature awaiting analysis. These samples were analyzed for total protein, globulin, albumin, albumin: Globulin ratio, total cholesterol, high density lipoprotein (HDL) cholesterol, high density lipoprotein (LDL) cholesterol and triglyceride in semi-automated analyzer by using diagnostic kits (Bayer Autopk biochemistry kits - Baroda) and methodology by manufacturer.

### Statistical analysis

For interpretation of results, the data obtained were subjected to statistical analysis as per Snedecor and Cochran [9].

## Results

### Growth performance

The effect of different levels of fat supplementation on growth performance is presented in the Table-3, 4 and 5. In the present study, significantly higher (p<0.05) body weight was recorded in birds fed 2.5% fat up-to 2^nd^ week, thereafter it was higher in 5% fat supplemented group till the end of the experiment as compared to others (Table-3). Similarly, significantly (p<0.05) higher feed intake was obtained due to 2.5% fat supplementation up to 2^nd^ week and afterwards no significant difference was recorded among treatments (Table-4). Birds fed 5% fat have improved FCR as compared to other group (p<0.05) on end of the ­experimental trial (Table-5).

**Table-3:** The effect of post hatch fat supplementation on body weight (g) of broiler chicken.

Particulars (%)	0 d	7 d	14 d	21 d	28 d	35 d
Control	43.26±0.33	153.76±1.76^a^	401.41±3.42^ab^	733.96±3.16^b^	1151.56±4.08^b^	1676.33±6.69^b^
2.5 fat	43.39±0.32	153.84±0.83^a^	405.98±3.74^a^	740.99±3.58^ab^	1161.96±5.06^ab^	1692.18±7.82^ab^
5 fat	43.60±0.72	151.09±0.82^b^	396.80±3.53^b^	744.97±3.40^a^	1166.99±4.59^a^	1698.32±9.13^a^
7.5 fat	45.20±0.57	145.92±1.36^c^	387.73±3.15^c^	734.78±3.88^b^	1153.16±6.54^b^	1678.76±8.19^b^
Significant	NS	**	**	*	*	*

Superscripts are read column wise for comparison of means. Means in the same column with different superscript a, b, c are significantly different

* (p <0.05) or

** (p <0.01), NS=Nonsignificant

**Table-4:** The effect of posthatch fat supplementation on feed intake (g) of broiler chicken.

Particulars (%)	7 d	14 d	21 d	28 d	35 d
Control	110.85±1.27^a^	445.94±2.82^a^	959.06±3.15	1753.00±5.09	2900.49±2.91
2.5 fat	110.90±0.57^a^	446.16±3.36^a^	953.41±3.62	1745.79±5.00	2889.98±6.24
5 fat	110.96±0.84^a^	437.51±3.67^ab^	949.34±3.87	1743.76±5.42	2879.10±6.43
7.5 fat	107.82±0.86^b^	434.88±1.27^b^	957.03±5.16	1741.62±7.18	2881.94±11.15
Significant	*	*	^NS^	^NS^	^NS^

Superscripts are read column wise for comparison of means. Means in the same column with different superscript a, b, c are significantly different

* (p <0.05) or (p**<0.01), NS=Nonsignificant

**Table-5:** The effect of posthatch fat supplementation on FCR of broiler chicken.

Particulars (%)	7 d	14 d	21 d	28 d	35 d
Control	0.72±0.00^b^	1.12±0.01^a^	1.31±0.01^a^	1.52±0.01	1.73±0.01^a^
2.5 fat	0.72±0.00^b^	1.10±0.01^b^	1.29±0.01^b^	1.50±0.01	1.71±0.01^a^
5 fat	0.73±0.01^a^	1.10±0.01^b^	1.27±0.01^b^	1.50±0.01	1.70±0.01^b^
7.5 fat	0.74±0.01^a^	1.12±0.01^a^	1.30±0.01^a^	1.51±0.01	1.72±0.01^a^
Significant	*	**	**	^NS^	**

Superscripts are read column wise for comparison of means. Means in the same column with different superscript a, b, c are significantly different

* (p <0.05) or

** (p <0.01), NS=Nonsignificant

### Carcass yield

Significantly (p<0.05) higher dressed weight was observed due to 5% fat supplementation than other groups, whereas, weight of other carcass cuts such as liver, heart, breast, thigh, back and neck and giblet was nonsignificant (Table-6).

**Table-6:** The effect of posthatch fat supplementation on carcass cuts (g) of broiler chicken.

Particulars (%)	Dressed wt	Liver	Heart	Gizzard	Breast	Thigh	Wing	Back and neck	Giblet
Control	1238.50±34.93	42.67±1.36	14.83±2.20	37.17^b^±0.44	171.00±3.91	340.50±13.03	333.83^ab^±19.32	287.83±1.36	94.67±2.73
2.5 fat	1280.50±43.56^ab^	42.75±1.79	14.33±1.28	36.42±0.44^b^	181.25±8.14	345.17±13.15	349.92±16.98^ab^	298.50±9.85	93.50±2.89
5 fat	1343.58±36.72^a^	44.42±2.23	12.67±1.58	42.25±2.08^a^	186.67±6.29	361.83±8.92	370.00±12.32^a^	314.58±12.46	99.33±5.19
7.5 fat	1212.67±20.86^b^	38.00±1.35	12.42±0.42	35.17±1.75^b^	173.25±7.31	324.00±11.26	319.25±6.40^b^	299.50±7.33	85.58±2.80
Significant	*	NS	NS	*	NS	NS	*	NS	NS

Superscripts are read column wise for comparison of means. Means in the same column with different superscript a, b, c are significantly different

* (p <0.05) or (p**<0.01), NS=Nonsignificant

### Protein and lipid content

**Figure-1:**
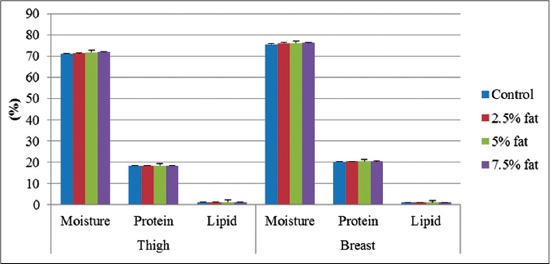
The effect of post hatch supplementation on moisture, protein and lipid content (%) of thigh and breast meat

No significant differences were observed in moisture, protein and lipid content of breast and thigh muscle of broiler due to fat supplementation (Figure-1).

### Immune response

The mean serum HI titer against New castle disease virus in the broilers on 28^th^ days showed significantly (p<0.05) difference due to fat supplementation (Table-7) and being higher in 2.5% fat supplemented group, whereas, on 35^th^ days it was nonsignificant.

**Table-7:** The effect of posthatch fat supplementation on serum HI titre (log2) against New Castle Disease virus of four week and five week old broiler chicken.

Particulars (%)	28 d	35 d
Control	2.11±0.22^c^	3.06±0.47
2.5 fat	3.33±0.24^a^	3.28±0.27
5 fat	2.72±0.25^ab^	3.83±0.25
7.5 fat	2.39±0.16^c^	3.25±0.21
Significant	*	NS

Superscripts are read column wise for comparison of means. Means in the same column with different superscript a, b, c are significantly different

* (p <0.05) or (p**<0.01), NS=Nonsignificant

### Biochemical profile

Higher serum albumin (g/dl) was recorded due to 5% fat supplementation whereas serum protein, globulin, A/G ratio, cholesterol, HDL cholesterol, LDL cholesterol and triglyceride did not showed any significance difference among treatments (Table-8).

**Table-8:** The effect of post hatch fat supplementation on organ weight factor of broiler chicken.

Particulars	Protein (g/dl)	Albumin (g/dl)	Globulin (g/dl)	A/G ratio	Cholesterol (mg/dl)	HDL cholesterol (mg/dl)	LDL cholesterol (mg/dl)	Tri-glyceride (mg/dl)
Control	3.73±0.23	1.56±0.14^b^	2.17±0.10	0.72±0.03	133.36±16.97	84.73±6.65	32.63±6.26	155.95±2.27
2.5 fat	3.83±0.17	1.61±0.08^b^	2.22±0.13	0.74±0.05	132.53±9.46	87.57±3.50	30.63±5.52	139.73±9.00
5 fat	4.09±0.11	1.87±0.04^a^	2.22±0.08	0.84±0.02	146.73±9.53	96.59±4.40	32.97±4.21	129.26±8.11
7.5 fat	3.93±0.08	1.69±0.04^ab^	2.24±0.06	0.76±0.02	164.26±11.26	94.33±6.66	42.27±3.96	135.46±8.98
Significant	NS	*	NS	NS	NS	NS	NS	NS

Superscripts are read column wise for comparison of means. Means in the same column with different superscript a, b, c are significantly different

* (p <0.05) or (p**<0.01), NS=Nonsignificant, LDL=Low density lipoprotein, HDL=High density lipoprotein

## Discussion

Fats are a concentrated form of energy and essential fatty acid which is affected by various factors including fat composition, age, species, gut status and diet composition. Younger birds digest fats less efficiently than the adult one, which is correlated with inadequate bile salt formation inside the body. Fats and oils are commonly added to poultry diets to ­p­roducing high-energy and nutrient concentrated formulations. Apart from that fat also influence the immune response of individuals. The respiratory quotient in hatching chicks in the range of 0.72-0.74 [10], indicating utilization principally of fat.

This study was designed to evaluate the effect of feeding four levels of fat on the growth performance, carcass characteristics and biochemical profile of broiler chickens. Broiler chicks that were fed 5% fat showed a significantly (p<0.05) improved FCR and higher body weight than other treatment groups at the end of the experimental trial. Similarly, significantly (p<0.05) higher feed intake was obtained due to 2.5% fat supplementation upto 2^nd^ week and afterwards no significant difference was recorded among treatments. The results of present finding were in accordance with finding of earlier workers [11,12]. Ghazalah *et al*. [11] observed an increased feed intake when birds supplemented with 5% fat during early week of post hatch, which might be due to increased absorption of fat, whereas other hydrophilic compounds such as glucose and amino acids are not well absorbed in yolk rich medium [1]. The observation after 1^st^ week in regard to feed intake were corroborated with findings of Soren *et al*. [13] who reported no positive effect due to fat supplementation during the starter and finisher stage. In contrast Tabiedian *et al*. [14] observed significant difference during the finisher phase. Ali *et al*. [15] have reported that feed intake decreased when broilers fed more than 7% fat in the diet, which is similar to present findings.

The major concern of the poultry industry is towards obtaining a higher dressing percentage and consequently increases the net profit. On the other hand, consumers are concerned about meat quality in terms of high nutritive value with lower fat percentage [16] as higher intake of saturated fatty acids causes an increase in serum cholesterol and subsequently, increases the risk of heart diseases. In the current study significantly (p<0.05) higher dressed weight was observed in birds fed 5% fat whereas, weight of other carcass cuts was nonsignificant among treatments except gizzard and wing. Ali *et al*. [12] also reported higher dressed weight due to fat supplementation in broilers diet.

To fulfill the consumer demand of healthier meat with lower fat percentage, there is a need of changing the fatty acid profile in the meat through suitable dietary manipulation [17]. Rezaei and Monfaredi [18] reported significantly higher lipid content of breast and thigh muscle due to fat supplementation, which was in complete disagreement with our findings where no significant differences were observed in moisture, protein and lipid content of breast and thigh muscle of broiler due to fat supplementation.

In current experiment higher serum albumin (g/dl) was recorded due to 5% fat supplementation, whereas other biochemical constituents did not show any significance difference among treatments. Current findings are supported by Burlikowska *et al*. [19] who reported that fat did not influence significantly the level of fat metabolism indices.

The present data of lipid profiles due to fat supplementation was not similar to the earlier findings of Monfaredi *et al*. [20] and Sahito *et al*. [21] who reported increased cholesterol, HDL and LDL due to supplemented fat.

## Conclusion

The results indicated that supplementation of fat significantly increased the body weight and improved FCR and dressing percentage.

## Author’s Contributions

KPR carried out the experiment. MKG and SPT designed and guided the experiment. TS and SKN help in sample processing and data analysis. All authors read and approved the final manuscript.
